# Genome-Wide Identification and Expression Analysis of the YTH Domain-Containing RNA-Binding Protein Family in *Cinnamomum camphora*

**DOI:** 10.3390/ijms25115960

**Published:** 2024-05-29

**Authors:** Jingjing Zhang, Sheng Yao, Xiang Cheng, Yulu Zhao, Wenya Yu, Xingyue Ren, Kongshu Ji, Qiong Yu

**Affiliations:** 1 State Key Laboratory of Tree Genetics and Breeding, Nanjing Forestry University, Nanjing 210037, China; ksji@njfu.edu.cn (K.J.); 2 Key Open Laboratory of Forest Genetics and Gene Engineering of National Forestry & Grassland, Nanjing Forestry University, Nanjing 210037, China; 3 Co-Innovation Center for Sustainable Forestry in Southern China, Nanjing Forestry University, Nanjing 210037, China; 4 Beijing National Laboratory for Molecular Sciences, Beijing 100190, China

**Keywords:** *N^6^*-methyladenosine (m^6^A), YTH domain, RNA-binding protein, *C. camphora*, *CcYTHs*

## Abstract

*N^6^*-methyladenosine (m^6^A) is one of the most abundant chemical modifications on mRNA in eukaryotes. RNA-binding proteins containing the YT521-B (YTH) domain play crucial roles in post-transcriptional regulation of plant growth, development, and stress response by reading the m^6^A mark. However, the YTH domain-containing RNA-binding protein family has not been studied in a valuable and medicinal tree such as *Cinnamomum camphora* (*C. camphora*) yet. In this study, we identified 10 *YTH* genes in *C. camphora*, located on eight out of 12 chromosomes. Phylogenetic analysis revealed that these genes can be classified into two major classes, *YTHDF* (*CcDF*) and *YTHDC* (*CcDC*). Closely related *CcYTHs* within the same class exhibited a similar distribution of conserved motifs and domain organization, suggesting functional similarities among these closely related *CcYTHs*. All CcYTH proteins possessed a highly conserved YTH domain, with *CcDC1A* containing an additional CCCH domain. The liquid–liquid phase separation (LLPS) predictions indicate that *CcDC1A*, *CcDF1A*, *CcDF1C*, *CcDF3C*, *CcDF4C*, and *CcDF5C* may undergo phase transitions. Quantitative expression analysis revealed that tissue-specific expression was observed fo *CcYTHs*. Notably, there were two genes, *CcDF1A* and *CcDF5C*; both exhibited significantly higher expression levels in various tissues than other genes, indicating that the m^6^A-YTH regulatory network in *C. camphora* might be quite distinct from that in most plants such as *Arabidopsis thaliana* (*A. thaliana*) with only one abundant YTH protein. According to the analysis of the up-stream cis-regulatory elements of these *YTH* genes, these genes could be closely related to stress, hormones, and development. The following stress response experiments further verified that their expression levels indeed changed under both PEG and NaCl treatments. These findings not only provide a foundation for future functional analysis of *CcYTHs* in *C. camphora*, but also provide insights into the functions of epigenetic mark m^6^A in forest trees.

## 1. Introduction

To date, over 170 types of RNA modifications have been reported [1]. *N^6^*-methyladenosine (m^6^A), as one of the most abundant RNA modifications in eukaryotes, plays a crucial role in gene regulation and maintenance of genomic stability [2]. Recent research has highlighted the diverse functions of m^6^A modification in RNA metabolism, such as splicing, translation, stability, and decay, thereby regulating various biological processes including gene expression, cell differentiation, embryonic development, and sex determination, as well as diseases [3,4,5]. Like for mammals, m^6^A is also important and necessary for plants, which can be dynamically written, erased, and read by m^6^A methyltransferases, demethylases, and m^6^A-binding proteins. In plants, MTA (homolog of human METTL3), MTB (homolog of human METTL14), VIR (homolog of human VIRMA), HAKA (homolog of human ZC3H13), FIP (homolog of human WTAP), and FIONA1 (homolog of human MELLT16) have been identified as writers of m^6^A [6,7,8,9]. ALKBH9B, ALKBH10B, and SlALKBH2 (homolog of human ALKBH5) have been characterized as m^6^A demethylases [10,11]. The readers have been named as the evolutionarily conserved C-terminal region (*ECT*) family proteins (homolog of human YTH-domain family proteins) [12]. The function of m^6^A modification depends mainly on its readers to specifically recognize the m^6^A marks on RNA to determine the RNA fate. Therefore, the identification of m^6^A readers is crucial for understanding the regulatory mechanisms of m^6^A on various physiological and molecular processes in cells.

The YTH protein family, a class of RNA-binding proteins that contains the YTH domain, has been first reported to serve as an m^6^A reader [13]. In 1998, YTH521, a member of the YTH protein family, was reported to function as an RNA splicing-related protein in rats [14]. In 2002, a novel YTH domain consisting of approximately 150 amino acids rich in aromatic residues was discovered within nuclear proteins and named as the YTH521-B homologous domain, which is a highly conserved in eukaryotes [15]. This domain folds into a conserved α/β fold, which includes 3α helices and 6β strands. The 6β strands form a barrel-like fold, and the 3α helices are packed against the β strands to create a hydrophobic core [16,17]. Within the YTH domain, an aromatic cage forms a hydrophobic binding pocket that is capable of recognizing buried methyl groups with a cavity insertion mode. This complex structure lays the foundation for the specific m^6^A recognition of YTH domain. Similar to most RNA-binding domains that are surrounded by structured domains or low-complexity regions with diverse functions, the YTH domain is also encircled by several disordered regions [18]. These disordered regions are indispensable for YTH family proteins to modulate the function of m^6^A-modified RNA by influencing the subcellular localization of YTH family proteins and their partners. Furthermore, these regions also impact their potential for liquid–liquid phase separation [19]. However, the study of m^6^A readers in plants remains limited. So far, only ECT2/3/4 and CPSF30-L, out of the 13 YTH-domain proteins that mainly come from the ECT family, have been identified as m^6^A readers in *A*. *thaliana*; the rest still remain unknown [20,21,22,23]. In vitro and in vivo experiments have confirmed that ECT2 could directly bind m^6^A, and its m^6^A binding activity is essential for normal trichome morphology [20]. Further research indicates that the functions of ECT2 are redundant with those of ECT3 and ECT4, suggesting that ECT3 and ECT4 also act as m^6^A readers [24,25]. CPSF30-L, the long isoform of the polyadenylation factor CLEAVAGE AND POLYADENYLATION SPECIFICITY FACTOR30 (CPSF30), consists of CPSF30-S and a YTH domain. This protein has been identified as a novel m^6^A reader in *A. thaliana*, and is the homolog of YTHDC1 located in the nucleus. It has been reported that CPSF30-L play a vital role in flowering transition and abscisic acid (ABA) response via recognizing m^6^A-modified RNA to enhance liquid–liquid phase separation and the formation of CPSF30-L nuclear bodies, then regulating alternative polyadenylation (APA) and affecting mRNA degradation, thereby regulating the flowering transition and ABA hypersensitivity, which provides a new insight into m^6^A regulation in plant RNA metabolism and phase separation [19,26]. According to the current various studies on *A. thaliana* and other plant species, it has been revealed that YTH proteins play vital roles in many biological processes including trichome branching, flowering transition, vegetative growth, reproductive development, nitrate signaling, and abiotic and biotic stresses. Consequently, it is necessary to study the YTH proteins in plants for better understanding the regulatory mechanisms of m^6^A on plant growth and development.

*C. camphora* is an important economic tree species with multiple applications in wood collection, medicine, fragrance, and ecology. As its branches and leaves are rich in essential oils and its seeds are abundant in medium carbon chain fatty acids, it is an important resource for camphor, linalool, natural flavors and medium carbon chain fatty acids. Therefore, it is worth it to study the function of m^6^A in such valuable tree. However, no m^6^A readers, the YTH domain-containing RNA-binding protein family, have been studied in *C. camphora* yet. In this study, we focused on the camphor tree and identified 10 *CcYTH* genes at the high-quality chromosome level in its genome through analyzing their chromosome localization, phylogenetic analysis, conserved motifs, gene structure, and cis-regulatory elements in the promoter regions. Additionally, the expression patterns of these *CcYTH* genes as well as their responses to stress treatments were also investigated. According to the results of these analysis, this study aimed to provide insights into the potential roles of *CcYTH* genes in the growth and development of *C. camphora*, which might bring a new viewpoint, from an epigenetic perspective, to research on this important tree breed.

## 2. Results

### 2.1. Identification and Phylogenetic Analysis of the C. camphora YTH Gene Family

**Figure 1:**
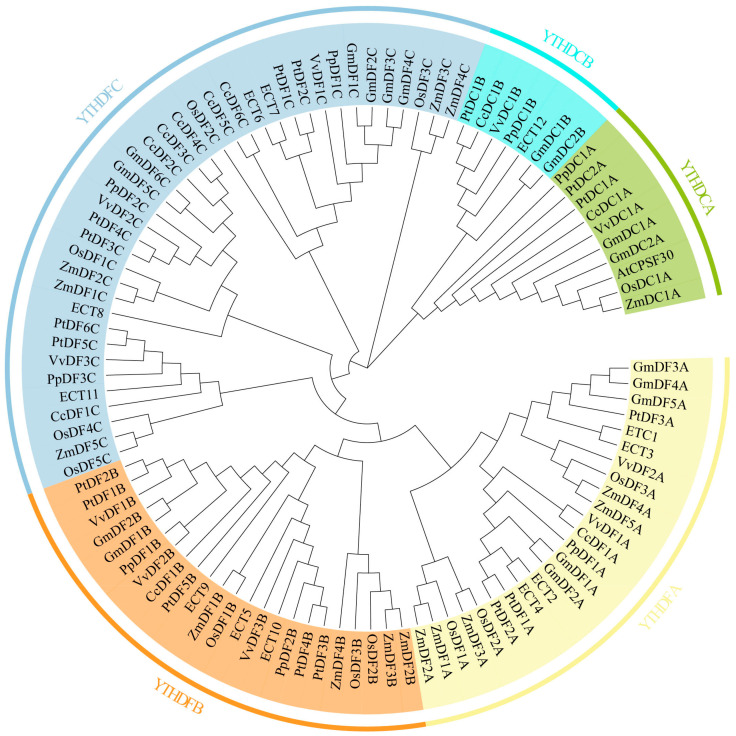
Phylogenetic tree of *CcYTH* genes. Different colored backgrounds and surrounding letters represent different groups. The phylogenetic tree resolved *YTH* genes into five groups. The YTH domain sequences are shown in Table S2.

Through HMMER searches, we ultimately identified 10 *CcYTH* genes from the camphor tree genome. (Figure 1; Table 1 and Table S1). In order to analyze the phylogenetic relationship of the CcYTH proteins in *C. camphora* and obtain the classification of the camphor tree *YTH* gene family, the neighbor-joining (NJ) method of MEGA 11 software was used to construct the phylogenetic tree of 102 YTH domain proteins from *A. thaliana* (13 members), *Glycine max* (*G. max*) (17 members), *Zea mays* (*Z. mays*) (15 members), *Oriza sativa* (*O. sativa*) (12 members), *Populus trichocarpa* (*P. trichocarpa*) (17 members), *Vitis vinefera* (*V. vinefera*) (10 members), *Prunus persica* (*P. persica*) (eight members), and *C. camphora* (10 members).The phylogenetic analysis showed that the *YTH* gene can be divided into two major groups: *YTHDF* (*CcDF*) and *YTHDC* (*CcDC*). The *YTHDF* family is composed of three subgroups: *YTHDFA*, *YTHDFB*, and *YTHDFC*, with one, one, and six members, respectively. The *YTHDC* family is divided into two subgroups: *YTHDCA* and *YTHDCB*, each with one member.

### 2.2. Synteny Analysis of the CcYTH Genes

**Figure 2:**
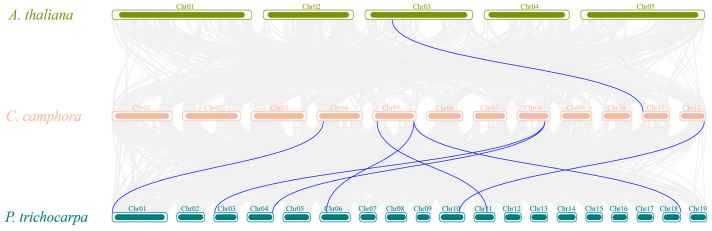
Synteny analysis of the *CcYTH* genes. Synteny analysis of *YTH* genes between *C. camphora*, *A. thaliana* and *P. trichocarpa*. Gray lines in the background indicate the collinear blocks within the genomes of *C. camphora* and other plants, and the blue lines indicate the syntenic *YTH* gene pairs.

To further infer the evolutionary mechanism of the *CcYTH* family in *C. camphora*, we constructed a collinearity analysis map of *C. camphora*, *A. thaliana*, and *P. trichocarpa*. The results showed that only one *CcYTH* gene exhibited collinearity with *A. thaliana*, while seven genes demonstrated collinearity with *P. trichocarpa* (Figure 2). This indicates that the *CcYTH* genes family has a closer evolutionary relationship with *P. trichocarpa* than with *A. thaliana*.

### 2.3. Protein Features and Gene Structure of the CcYTH Genes

**Figure 3:**
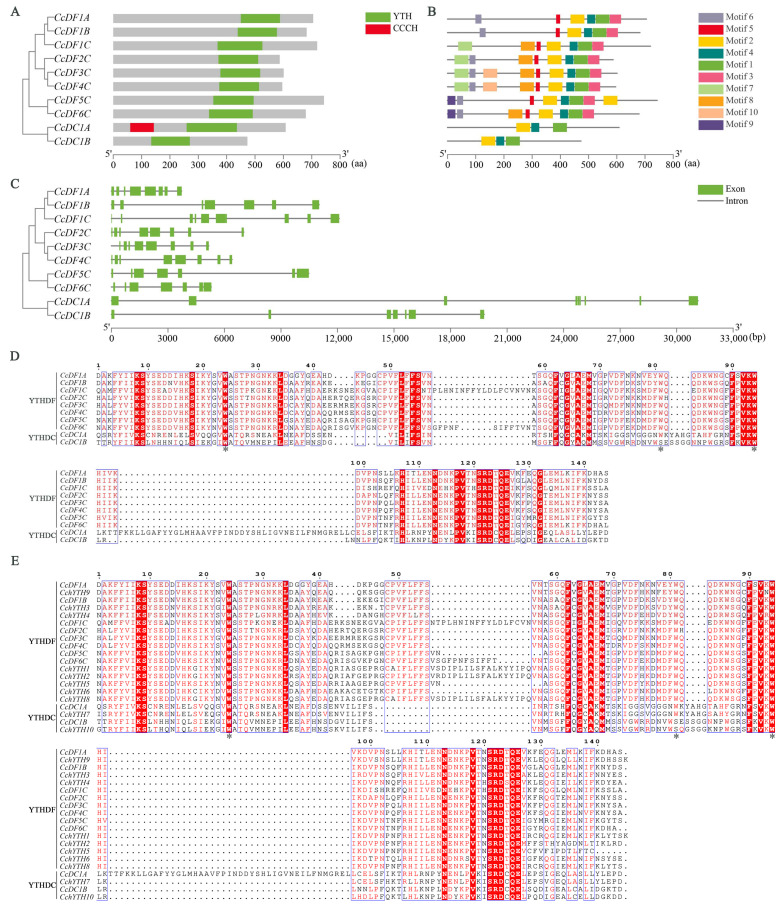
Conserved YTH domains, motifs, and gene structures of CcYTH proteins. (**A**) YTH domains of CcYTH proteins. (**B**) Distributions of conserved motifs in CcYTH proteins. Ten putative motifs are indicated in different colored boxes. A detailed description of the 10 motifs is provided in Supplementary Materials. (**C**) Exon/intron organizations of *CcYTH* genes. Green boxes represent exons, and black lines represent introns. (**D**) Sequence comparison of YTH domain of CcYTH proteins. The position of tryptophan is indicated by an asterisk. (**E**) Sequence comparison of YTH domain of CcYTH and CchYTH proteins. The position of tryptophan is indicated by an asterisk.

The physicochemical properties of the CcYTH proteins were calculated using the ProtParam software (Table 1). The full length of the CcYTH proteins ranges from 473 amino acids (*CcDC1B*) to 743 amino acids (*CcDF5C*), with a molecular weight of 53.5 kDa (*CcDC1B*) to 81.29 kDa (*CcDF5C*) and an isoelectric point of 5.21 (*CcDF4C*) to 8.80 (*CcDF1C*). The subcellular localization prediction results indicated that, except for *CcDF1C*, *CcDF5C*, and *CcDF6C*, the other seven CcYTH proteins are likely predominantly localized in the nucleus. To further analyze the structural diversity of CcYTH proteins, the gene structure, conserved domain, and motifs of CcYTH proteins were analyzed. From the analysis of conserved domain, the results showed that a typical functional YTH domain exists in each CcYTH proteins and that the b-type *YTHDC* (*CcDC1B*) lacks any additional structural domains apart from the YTH domain (Figure 3A). Moreover, the N-terminus of *CcDC1A* in camphor trees contains a CCCH domain, while that of *CcDC1B* does not. According to the prediction of the CcYTH proteins motif performed by using the MEME tool, a total of 10 conserved motifs were identified from the YTH proteins of camphor trees, and motifs 1, 2, and 4 were present in all CcYTH proteins (Figure 3B and Table S3). Moreover, similar distribution of conserved motifs has been observed within the same group of CcYTH proteins, indicating that they may have similar functions within same group. Gene structure analysis showed that all members of the *CcYTH* genes family belong to split genes containing at least seven introns (Figure 3C). Members of the *CcYTH* genes family exhibit completely different gene structures in terms of the size and arrangement of exons and introns, indicating the independent evolution of these genes. Interestingly, in the gene structure of most *YTHDF* subfamily members, there are two longer exon segments in the middle position. It is worth noting that in both *C. camphora* and *C. chekiangoleosa*, except for the second tryptophan (W) residue replaced by serine (S) in *CcDC1B* of the YTHDC family and *CchYTH10*, all other aromatic cages of YTH consist of tryptophan residues (WWW) (Figure 3D,E).

### 2.4. The Interaction and Tertiary Structure Prediction of CcYTH Proteins

**Figure 4:**
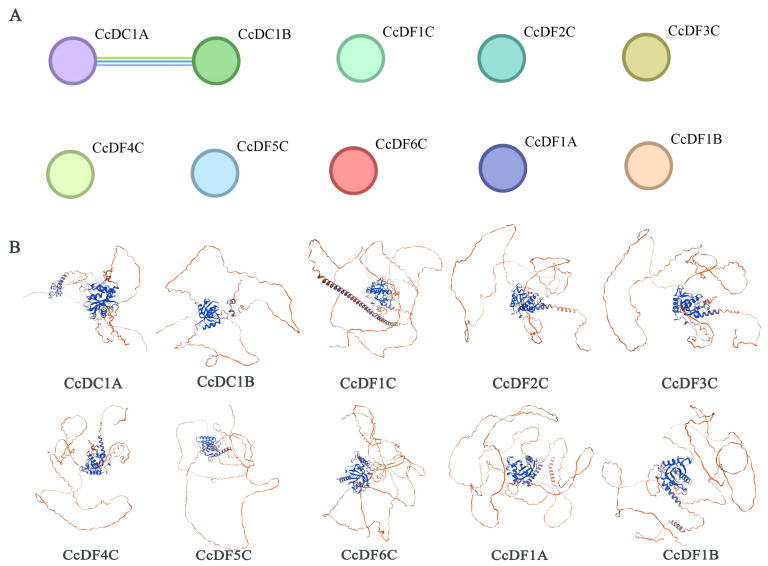
The interaction and three-dimensional structure of CcYTH proteins. (**A**) The protein–protein interaction of CcYTH proteins. (**B**) The tertiary structure of CcYTH family proteins.

To further understand the interaction relationships among CcYTH protein members, protein–protein interaction prediction was performed on the amino acid sequences of 10 members. The results show that there is only an interaction between CcDC1A and CcDC1B, while no interactions were found among the other CcYTH proteins (Figure 4A). However, the specific interaction network mechanism between CcDC1A and CcDC1B members is not clear and requires further investigation. Homology modeling was conducted on the amino acid sequences of 10 *C. camphora* YTH proteins. The results indicate that the majority of CcYTH protein sequences share highly similar three-dimensional structures, with higher similarity observed within subfamily members compared to those between subfamilies. Overall, these YTH domains typically adopt a specific mixed α-helix-β-sheet fold, where β-sheets are arranged into a β-barrel structure and surrounded by α-helices (Figure 4B). A well-defined conserved aromatic cage is observed in all YTH domains, endowing them with the ability to distinguish and recognize m6A-modified RNA.

### 2.5. Prion Subsequences Analysis of the CcYTH Proteins Family

**Figure 5:**
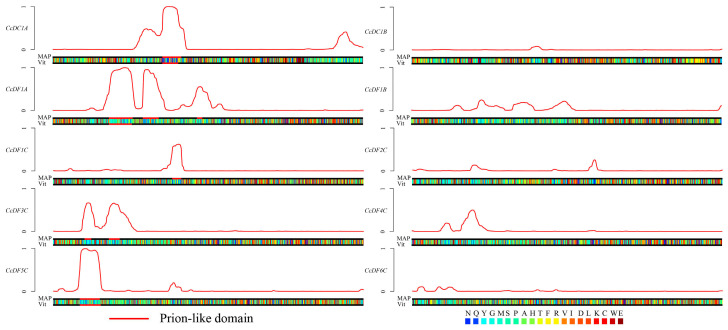
Identified probable prion subsequences of *CcYTH* genes family in *C. camphora*. The red line is the prediction of the prion structure region. If the red line is in the non-baseline region, it indicates that the prion structure region exists at that location and the phase transition is highly likely.

The disorder region of prion-like amino acid composition is a prerequisite for the protein to have phase transition ability [27]. By predicting whether YTH protein family members contain disorder regions, their potential for phase transition can be determined. PLAAC (Prion-Like Amino Acid Composition) is a powerful bioinformatics tool that has been used to identify prion-like domains in a wide range of proteins, including those involved in RNA binding, transcriptional regulation, and signal transduction [28]. Recent research has reported that some members of the *YTH* gene family in mammals or Arabidopsis can enhance the phase transition ability when binding to m^6^A sites [26,29]. The results showed that *CcDC1A*, *CcDF1A*, *CcDF1C*, *CcDF3C*, *CcDF4C*, and *CcDF5C* possess at least one the disorder region of prion-like amino acid composition, while no disorder region was found in the rest of CcYTH proteins, indicating these six CcYTH proteins might have the ability to undergo phase transition (Figure 5).

### 2.6. Tissue Expression Patterns of CcYTH Genes

**Figure 6:**
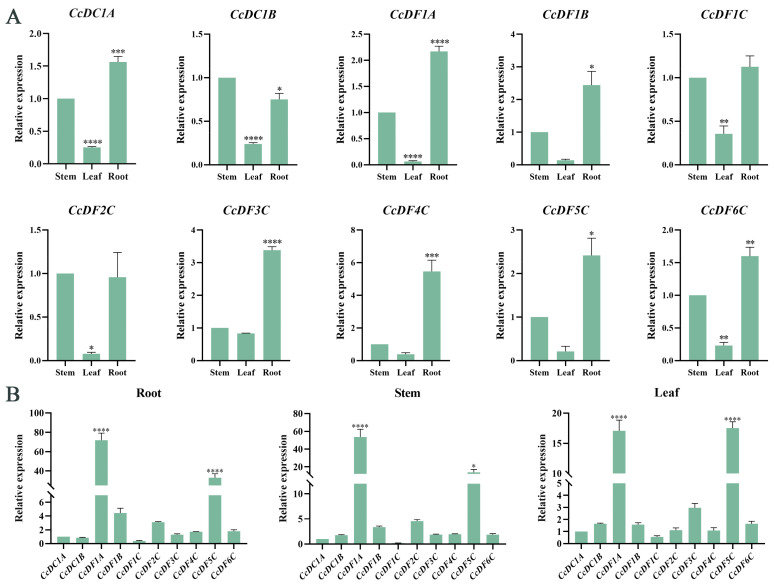
Tissue expression specificity analysis of *CcYTH* genes. (**A**) Expression patterns of *CcYTH* genes under different tissues. The relative expression in “Stem” was set as 1. (**B**) Expression patterns of different *CcYTH* genes in the same tissue. Using the expression of the *CcDC1A* gene as a control, the relative expression levels were determined. Each value represents mean  ±  standard error (SE) of three replicates. Asterisks indicate significant differences in transcript abundance compared to the control group (* *p* < 0.05, ** *p* < 0.01, *** *p* < 0.001, **** *p* < 0.0001).

To investigate the potential physiological roles of the *CcYTH* genes in the growth and development of *C. camphora*, the tissue-specific expression patterns of *CcYTH* genes surveyed across different tissues revealed distinct expression levels of the 10 genes in roots, stems, and leaves (Figure 6A). Interestingly, most genes exhibited the highest expression levels in roots, moderate expression levels in stems, and the lowest expression levels in leaves. Notably, *CcDF1A* and *CcDF5C* were expressed at much higher levels than other *CcYTH* genes in these three tissues (Figure 6B), which is quite different from the findings regarding other plants, such as *A. thaliana* and *C. chekiangoleosa*, with only one dominantly high-expressed *YTH* gene across all tissues. It indicates that *C. camphora* has a unique YTH regulatory network to achieve downstream biological functions of m^6^A modification, which is worthy of further investigation into the functions of these two genes.

### 2.7. Subcellular Localization of CcDF1A and CcDF5C Proteins

**Figure 7:**
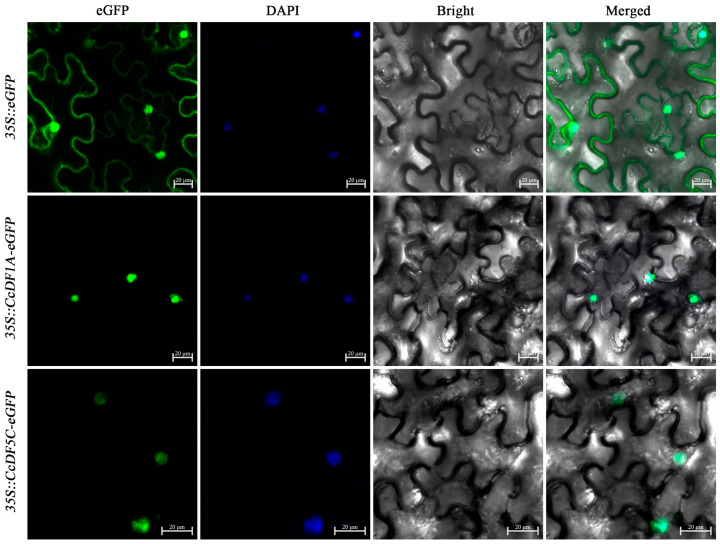
Subcellular localization experiments of *CcDF1A* and *CcDF5C* proteins. Transient expression of eGFP (control), *CcDF1A*, and *CcDF5C* eGFP in tobacco leaves. The scale bar in the images of is 20 μm.

The subcellular localization prediction results indicated that most of CcYTH proteins are likely predominantly localized in the nucleus. In order to verify the accuracy of these prediction, we used transient expression experiment on tobacco to investigate the subcellular localization of CcYTH proteins. Due to the high expression levels in the roots, stems, and leaves among these CcYTH genes, CcDF1A and CcDF5C were selected for subcellular localization experiments. The results showed that both CcDF1A and CcDF5C exhibit green fluorescence exclusively within the cell nucleus (Figure 7), which confirmed the predicted localization of these two proteins in the nucleus.

### 2.8. Analysis of Cis-Regulatory Elements in the Promoter Region of the CcYTH Genes

**Figure 8:**
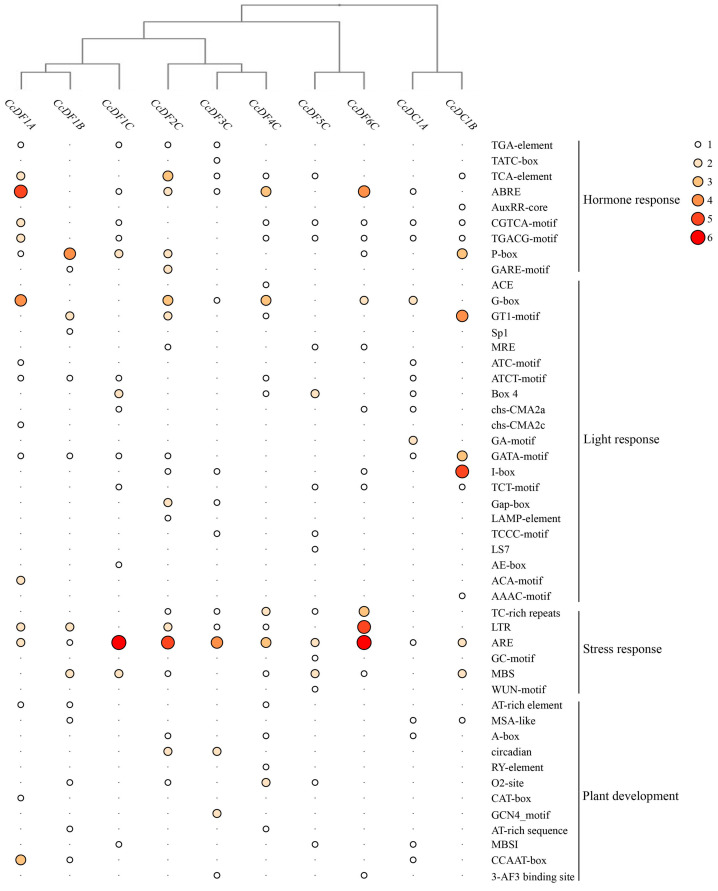
The cis-regulatory elements in the promoter of the *CcYTH* genes. The online software PlantCARE was used to analyze the 2 kb sequence upstream of the transcription start site of each *CcYTH* genes.

The promoter analysis can provide a deep understanding of gene function. In order to investigate the cis-regulatory elements in the promoter region of the *CcYTH* genes, 2.0 kb upstream sequences of each *CcYTHs* translation start site were analyzed. The results showed that there are a large number of stresses, hormone, and development-related cis-regulatory elements in the promoters of *CcYTHs* (Figure 8 and Table S4). The hormone-responsive elements include methyl jasmonate (MeJA)-responsiveness elements (*CcDC1A*, *CcDC1B*, *CcDF1A*, *CcDF1C*, *CcDF4C*, *CcDF5C*, and *CcDF6C*), abscisic acid responsiveness elements (*CcDC1A*, *CcDF1A*, *CcDF1C*, *CcDF2C*, *CcDF3C*, *CcDF4C*, and *CcDF6C*), auxin-responsive elements (*CcDC1B*, *CcDF1A*, *CcDF1C*, *CcDF2C*, and *CcDF3C*), salicylic acid responsiveness elements (*CcDC1B*, *CcDF1A*, *CcDF2C*, *CcDF3C*, *CcDF4C*, and *CcDF5C*), and gibberellin-responsiveness elements (*CcDC1B*, *CcDF1A*, *CcDF1B*, *CcDF1C*, *CcDF2C*, *CcDF3C*, and *CcDF6C*), and each gene promoter contains at least one plant hormone-responsive element. In addition to the plant hormone-related elements, several cis-regulatory elements involved in abiotic stress were also identified, including drought-inducibility (*CcDC1B*, *CcDF1B*, *CcDF1C*, *CcDF2C*, *CcDF4C*, *CcDF5C*, and *CcDF6C*), defense and stress-responsiveness (*CcDF2C*, *CcDF3C*, *CcDF4C*, *CcDF5C*, and *CcDF6C*), and low-temperature responsiveness (*CcDF1A*, *CcDF1B*, *CcDF2C*, *CcDF3C*, *CcDF4C*, and *CcDF6C*) elements. Circadian rhythm control elements were found in the promoters of *CcDF2C* and *CcDF3C*. Additionally, there are numerous elements related to light response. These elements can be categorized into four main classes: stress response, plant growth and development, light response, and hormone response elements. In summary, the presence of these cis-regulatory elements in the *CcYTHs* promoter suggests that the *CcYTHs* family may be involved in regulating plant responses to hormones and abiotic stresses in *C. camphora*.

### 2.9. Expression Patterns of CcYTH Genes under Stress Conditions

**Figure 9:**
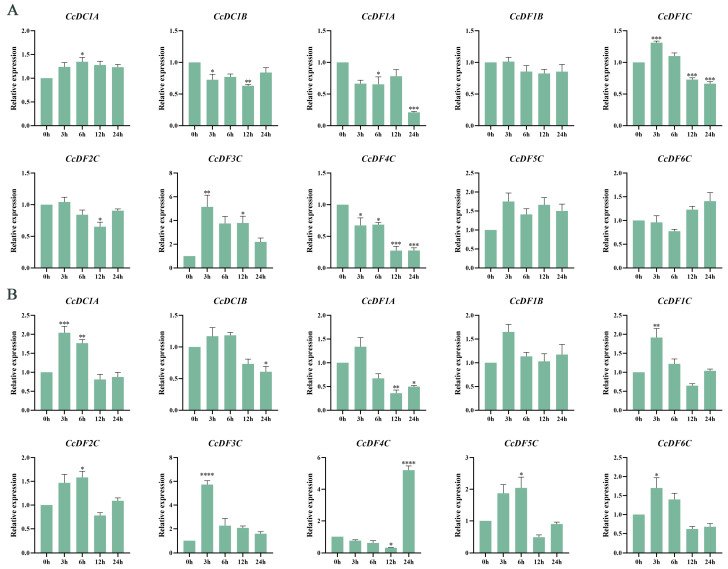
Expression Patterns of the *CcYTH* genes in Response to Abiotic Stresses. (**A**) Expression patterns of *CcYTH* genes under PEG stress. (**B**) Expression patterns of *CcYTH* genes under NaCl stress. For NaCl and PEG stress, leaves were sampled at 0 h, 3 h, 6 h, 12 h, and 24 h. Asterisks indicate significant differences in transcript abundance in the treated group compared to the control group (0 h) (* *p* < 0.05, ** *p* < 0.01, *** *p* < 0.001, **** *p* < 0.0001).

The promoter regions of 10 *CcYTH* genes in *C. camphora* contain a large number of elements related to stress response. To comprehend the response patterns of *YTH* genes under stress, we conducted drought and salt stress treatments on *C. camphora*. The results indicated that after PEG simulated drought treatment, *CcDC1A*, *CcDC1B*, *CcDF1A*, *CcDF1C*, *CcDF2C*, *CcDF3C*, and *CcDF4C* all exhibited significant changes in expression patterns within 24 h. Among them, the relative expression level of *CcDF3C* increased significantly at 3 h, showing a 5.1-fold change (Figure 9A). With the exception of *CcDF4C*, the expression of other genes showed an initial increase followed by a decrease within 0 to 24 h after NaCl treatment. In contrast, *CcDF4C* showed a decrease initially, followed by a highly significant upregulation in expression at 24 h (Figure 9B). Interestingly, under both PEG and NaCl treatments, the *CcDF3C* gene displayed a similar expression pattern of initially increasing and then decreasing, with its expression level increasing approximately 5-fold within 3 h. This gene exhibited the most significant upregulation among the 10 *CcYTH* genes.

## 3. Discussion

m^6^A modification is the most common post-transcriptional modification of RNA in eukaryotes, and it is extensively involved in biological processes such as gene regulation, post-transcriptional regulation, RNA metabolism, and disease occurrence [5]. In recent years, research on m^6^A in plants has uncovered that it plays an important role in plant growth, development, and stress response, making it become an important hotspot in plant molecular breeding and functional genomics research [3]. Revealing the molecular regulatory mechanisms of m^6^A in various organs or developmental processes is a promising direction for future research. The recognition of m^6^A sites by reader proteins is critical for executing the multifunctional roles of m^6^A modifications [12]. m^6^A readers determine the fate of their target RNAs, thereby exerting physiological impacts. Hence, investigating m^6^A readers could serve as a valuable entry point to explore how m^6^A modifications exert their influence on specific organs or biological processes [30].

It has been demonstrated that YTH family members function as m^6^A reader proteins [31]. These family proteins contain highly conserved YTH domains, which enable them to specifically bind to m^6^A modified RNA and participate in various biological processes. In humans, RNA-binding proteins with YTH domains have been extensively studied and five YTH proteins have been identified—YTHDC1, YTHDC2, YTHDF1, YTHDF2, and YTHDF3—that employ conserved mechanisms to recognize m^6^A modifications but lead to different fates of RNAs [2]. YTHDC1 mediates pre-mRNA splicing through interaction with splicing factors. YTHDC2 can regulate spermatogenesis in mammals. YTHDF1 interacts with the translation initiation machinery and enhances the translation efficiency of its target RNAs. YTHDF2 accelerates the degradation of m^6^A-modified transcripts and YTHDF3 can mediate mRNA decay by directly interacting with YTHDF2 [32,33,34,35,36].

Although there is extensive research on YTH proteins in animals, the study of YTH proteins and their functions in plants remains limited. YTH domain-containing RNA-binding proteins were initially identified in *A. thaliana* and *O. sativa*, with 13 and 12 members, respectively [23]. The biochemical studies on some YTH proteins found that recombinant AtYTH05 can bind to single-stranded RNA molecules, suggesting that AtYTH05 protein possesses RNA binding activity in vitro. This is similar to what has been previously demonstrated for YT521-B, which binds to single-stranded RNA sequences [13]. However, only the functions of ECT2/3/4 and CPSF30-L have been detailed and reported as m^6^A readers in regulating trichome morphology, vegetative growth, reproductive development, flowering transition, and abscisic acid (ABA) response in *A. thaliana*; the rest still remain unknown [19,20,21,22,26,37]. Therefore, the role of plant YTH proteins deserves further investigation. Compared to *A. thaliana* and *O. sativa*, a much larger number of *YTH* genes, 39 in total, have been identified in *Triticum aestivum* (*T. aestivum*) [38]. The aromatic cage in *TaDFs* is composed of tryptophan, tryptophan, and tryptophan (WWW), while in *TaDCs*, the aromatic cage consists of tryptophan, tryptophan, and tyrosine (WWY). In the genome of woody plant apple, a total of 26 putative *YTH* genes were identified [39]. Among them, nine *YTH* genes are involved in the senescence response of apple, and the expression of these genes is upregulated with leaf aging. So far, there are still many plant YTH proteins in the plant kingdom that have not been studied, especially in woody plants.

In the analysis of the camphor tree genome, a total of 10 YTH domain-containing proteins were identified, which have important effects on the growth and adaptation of *C. camphora*. Compared to the five *YTH* genes found in mammals, there are 10 *YTH* genes present in *C. camphora*, including one DFA subfamily, one DFB subfamily, six DFC subfamilies, one DCA subfamily, and one DCB subfamily, implying a more complex regulatory mechanism or functional redundancy among YTH family members. (Figure 1 and Table 1). For example, in *A. thaliana*, *ECT2*/*3*/*4* exhibit redundant functions [22]. Except for the presence of two *CcYTH* genes on chromosomes 04 and 05, most *CcYTH* genes are dispersed across different chromosomes (Table 1). Additionally, the two *CcYTH* genes on chromosomes 04 and 05 are located far apart, positioned at the ends of the chromosomes, indicating that the evolution of the *C. camphora CcYTH* genes family does not involve tandem duplication or segmental duplication events. The syntenic map of *C. camphora*, *A. thaliana*, and *P. trichocarpa* indicates that apart from *CcDF5C*, other *CcYTH* genes do not show collinearity with *A. thaliana*, whereas as many as seven *CcYTH* genes exhibit collinearity with *P. trichocarpa* (Figure 2). This suggests a closer evolutionary relationship between *C. camphora* and *P. trichocarpa* and similar functions of YTH proteins in woody plants compared to herbaceous plants, which indicates that it is necessary to investigate the m^6^A readers in woody plants to reveal the distinctive roles of m^6^A in the plant kingdom. The results of the structural diversity analysis of CcYTH proteins indicate the presence of a typical functional YTH domain in each CcYTH proteins. Similar conserved motifs are found in the same group of CcYTH proteins, while they exhibit completely different gene structures in terms of the size and arrangement of exons and introns, suggesting that these genes have evolved independently, and similar functions might be discovered in same group (Figure 3). The predicted interaction relationship among CcYTH proteins members indicates that only *CcDC1A* and *CcDC1B* interact with each other. The results of the tertiary structural homology modeling show that the YTH domains of CcYTH proteins typically adopt a specific mixed α-helix-β-sheet structure (Figure 4). The m^6^A modification can enhance the phase separation potential of mRNA [29]. Prion subsequences analysis of the CcYTH proteins family suggests that *CcDC1A*, *CcDF1A*, *CcDF1C*, *CcDF3C*, *CcDF4C*, and *CcDF5C* may undergo phase transition in its recognition of m^6^A to regulate plant biological processes (Figure 5).

Different plant organs have evolved different functions in plants. For example, root is the important organ for plants to absorb water and nutrients. Many genes have tissue and organ specific expression patterns in plants which can effectively regulate the growth and development as well as the response to stress of plants in time. The results of tissue-specific expression patterns of *CcYTH* genes in different tissues indicate that seven *CcYTH* genes exhibit significantly higher expression levels in roots than in other tissues (Figure 6), suggesting that *CcYTH* genes might have distinct potential functions in different tissue and play a significant role in the growth and development of roots in *C. camphora*. From the expression levels of *CcYTH* genes across all the tissues, it is obvious that the expression levels of *CcDF1A* and *CcDF5C* are higher than the others, indicating that they might hold a main position in the recognition of m^6^A among all the YTH domain containing m^6^A readers. This discovery that both these two YTH proteins including YTHDF protein and YTHDC protein are abundant in *C. camphora* is quite different from the research reported regarding Arabidopsis and other plants that possess only one YTH protein, usually YTHDF protein with highest expression, indicating the unique m^6^A regulatory network in *C. camphora*. Subcellular localization of protein can provide the possible transport mechanisms and their roles in cellular activities and signal transduction. The two genes with the highest expression levels in *C. camphora*, *CcDF1A* and *CcDF5C*, are both localized in the nucleus (Figure 7), which suggests that the m^6^A recognition process may primarily occur in the nucleus along with phase–phase separation to regulate the downstream physiological and biological progress in *C. camphora*. Hence, it is deserved to further uncover the regulatory mechanism of these m^6^A readers in *C. camphora*. The cis-regulatory element analysis of *C. camphora* promoters showed that the promoter regions of the 10 *CcYTH* genes contain numerous cis-regulatory elements associated with stress responses (Figure 8), which is similar to the findings in various plants such as Arabidopsis, rice, poplar, and so on [23,30,40,41,42]. The expression levels of genes related to development and stress response are generally high or significantly increased during the developmental stage or under stress treatments. With regard to the enrichments of cis-regulatory elements related to stress and the encountering threaten from drought or salinity to *C. camphora*, we took PEG and NaCl treatments into account to investigate the changes of these *CcYTH* genes after treatment to unveil the relationship between stress response and m^6^A. Under treatment with PEG and NaCl, the expression levels of the *CcYTH* genes exhibited different trends of variation, suggesting that each member of *CcYTH* genes family might have a specific function in response to stress (Figure 9).

While identifying the *YTH* genes family in *C. camphora*, we also identified the *YTH* genes family in *Camellia chekiangoleosa* (*C. chekiangoleosa*), one of the main cultivated tree species with a short maturation period, easy harvest, high oil content in seeds, and superior quality of tea oil [43]. According to the results, 10 randomly distributed *YTH* genes belonging to the five *YTHDFA*, *YTHDFB*, *YTHDFC*, *YTHDCA*, and *YTHDCB* subfamilies were identified in both *C. camphora* and *C. chekiangoleosa*. The collinearity analysis results show that compared to *A. thaliana*, both plants have a closer evolutionary relationship with *P. trichocarpa*. Conservative domain analysis results showed that each YTH protein contains a typical functional YTH domain. In both *C. camphora* and *C. chekiangoleosa*, apart from the second tryptophan (W) residue replaced by serine (S) in *CcDC1B* of the YTHDC family and *CchYTH10*, all other aromatic cages of YTH comprise tryptophan residues (WWW). Prion subsequences prediction results showed that there may be seven and nine *YTH* genes that might have the ability to undergo phase transition in *C. camphora* and *C. chekiangoleosa*, respectively. Analysis of tissue-specific expression results showed that the expression levels of genes in the *YTHDFA* subfamily (*CcDF1A* and *CchYTH9*) in *C. camphora* and *C. chekiangoleosa* were significantly higher in all tissues compared to other genes. Interestingly, unlike oil tea, there is also a gene, *CcDF5C*, whose expression level is significantly higher than other genes except for *CcDF1A* in *C. camphora*. Analysis of cis-regulatory elements in the promoter regions showed that both *C. camphora* and *C. chekiangoleosa* contain a large number of elements related to hormone, light, stress, or plant growth and development response in their promoter regions. When facing stress, most *YTH* genes in both *C. camphora* and *C. chekiangoleosa* showed significantly upregulated or downregulated expression levels. This indicates that *YTH* genes play a positive regulatory role in plants under stress conditions.

All these findings demonstrate the important and special roles of m^6^A in the developmental and stress responsive processes of *C. camphora* as well as the predicted distinctive and unique m^6^A-YTH regulatory mechanism in *C. camphora*.

## 4. Materials and Methods

### 4.1. Plant Materials and Treatments

Two-year-old camphor seedlings were provided by the Jiangxi Provincial Engineering Research Center for Seed-breeding and Utilization of Camphor Trees of Nanchang Institute of Technology. They were cultivated in nutrient soil under conditions of 25 °C and a 16 h light/8 h dark cycle at the State Key Laboratory of Tree Genetics and Breeding, Nanjing Forestry University (N 32°04′43.05″, E 118°49′1.70″). Fresh root, stem, and leaf samples were collected for tissue-specific expression analysis. For stress experiments, 100 mM NaCl solution and 20% polyethylene glycol (PEG6000) solution were used to water the soil. Leaf samples were collected from each treated seedling at 0 h, 3 h, 6 h, 12 h, and 24 h after the stress was applied, along with untreated leaf samples at 0 h as a control. The collected samples were immediately frozen in liquid nitrogen and stored at −80 °C for subsequent RNA extraction. All treatments were carried out in three biological replicates.

### 4.2. Identification of the YTH Genes in C. camphora

To identify genes containing the YTH domain in *C. camphora*, the gene annotation and genome files of *C. camphora* were downloaded from the National Genomics Data Center (https://ngdc.cncb.ac.cn/, accessed on 27 March 2023) [44]. Subsequently, the Hidden Markov Model (HMM) from the HMMER 3.0 program was employed to search for the YTH521-B domain (PF04146) within the *C. camphora* genome database, with a cutoff e-value of 1 × 10^−5^ [45]. The amino acid properties, molecular weight (MW), aliphatic index, grand average of hydropathicity (GRAVY) and isoelectric point (pI) were determined using the ProtParam tool (http://web.expasy.org/protparam/, accessed on 27 March 2023). The subcellular localizations were predicted by using the Cell-PLoc online tool (http://www.csbio.sjtu.edu.cn/bioinf/Cell-PLoc/, accessed on 28 March 2023).

### 4.3. Phylogenetic Analysis, Chromasomal Location and Synteny Analysis

To investigate the phylogenetic relationships among YTH proteins, the YTH protein sequences of *A. thaliana, G. max, Z. mays, O. sativa*, *P. trichocarpa*, *V. vinefera*, *P. persica*, and *C. camphora* were compared. The phylogenetic tree was generated using MEGA 11 software (https://megasoftware.net/, accessed on 29 May 2023) with neighbor-joining (NJ) method for adjacency, Jones–Taylor–Thornton (JTT) model, pairwise deletion and bootstrap (1000 repetitions). The protein sequences and their gene names (ID) are listed in Supplementary Materials Table S1. The chromosomal location information of the *CcYTH* genes was extracted from the camphor tree GFF file. Then, TBtools (https://github.com/CJ-Chen/TBtools/releases, accessed on 30 May 2023) were utilized for generating the chromosome position map of the *CcYTH* genes. For synteny analysis, a homology analysis map was constructed by using TBtools.

### 4.4. Gene Structure, Conserved Motifs and Consevered Domain Analyses

Gene structure analysis was performed to identify exons and introns. The corresponding GFF data were extracted from the GFF file in *C. camphora* Genome Database. Then, the gene structure was analyzed and visualized by TBtools. The conserved motifs of *CcYTH* proteins were analyzed using MEME (https://meme-suite.org/meme/, accessed on 31 May 2023). The maximum number of protein motifs was set to 10, the length of the motifs ranged from 15 to 50, and the output MEME file was further modified with TBtools. The domains of CcYTH proteins were analyzed using Conserved Domains Database (CDD) (https://www.ncbi.nlm.nih.gov/Structure/cdd/wrpsb.cgi, accessed on 31 May 2023).

### 4.5. Identification of Protein-Protein Interactions, Tertiary Structures, and Prion-like Subsequences of the CcYTH Proteins Family

We used STRING (https://string-db.org/, accessed on 17 May 2024) for predicting the interactions among the *C. camphora* YTH protein members, with *Cinnamomum micranthum*, a member of the same Lauraceae family, selected as a reference. The protein’s three-dimensional structure models were established using the SWISS-MODEL (https://swissmodel.expasy.org/, accessed on 17 May 2024). The *PLAAC* online website (http://plaac.wi.mit.edu/, accessed on 1 June 2023) uses Hidden Markov Model (*HMM*) algorithm to search CcYTH proteins sequences to identify possible prion sub-sequences [46]. The minimum length of the hidden Markov model for the prion-like domain is set to 60, with a background frequency of 100%.

### 4.6. Cis-Regulatory Elements Analysis of the CcYTH Genes Promoter

The 2000 bp genomic sequence upstream of transcription start site of each gene was chosen as its promoter sequence. The cis-regulatory elements in the region of promoter were identified using PlantCARE (https://bioinformatics.psb.ugent.be/webtools/plantcare/html/, accessed on 3 June 2023), and then visualized using TBtools.

### 4.7. Subcellular Localization of CcDF1A and CcDF5C Proteins

The primers CcDF1A-F, CcDF1A-R, and CcDF5C-F, CcDF5C-R were used to amplify the coding sequences of CcDF1A and CcDF5C, respectively (Table S5). The coding region of CcDF1A and CcDF5C was cloned into the pCAMBIA1305 vector between XbaI and SalI restriction sites with C-terminal eGFP, driven by the CaMV35S promoter, respectively. The vectors carrying p35S:: CcDF1A-eGFP and p35S:: CcDF5C-eGFP were introduced into Agrobacterium cells EHA105, which co-cultured with the p19 (RNA silencing suppressor) according to previous report [47]. Agrobacterium strain was infiltrated with a syringe into the leaves of Nicotiana benthamiana for transient expression. Two days later, the GFP signals in the infiltrated areas were captured using a confocal microscope (LSM710, Zeiss, Jena, Germany) at an excitation wavelength of 488 nm, while the DAPI signals were imaged at an excitation wavelength of 405 nm.

### 4.8. Total RNA Extraction, and Expression of the CcYTH Genes Analyzed by qRT-PCR

The total RNA samples were extracted from the tissues of two-year-old *C. camphora* according to the manufacturer’s protocol by using the Polysaccharides and Polyphenolics-rich Plant Total RNA Isolation Kit (Vazme Biotechnology, Nanjing, China). The concentration and purity of total RNA were measured using a Nano-Drop 2000 (Thermo Fisher Scientific, Waltham, MA, USA), and its integrity was confirmed through 1.2% agarose gel electrophoresis. The cDNA was synthesized from 1 µg of total RNA using the 1st Strand cDNA Synthesis Kit (Yeasen Biotechnology, Shanghai, China).

The qPCR reactions were carried out via a StepOne Plus Real-Time PCR System (Applied Biosystems, Foster City, CA, USA). The reaction mixture was as follows: 1 µL of cDNA after 20-fold dilution, 0.4 µL of each forward and reverse primer (10 µM), 10 µL of SYBR Green Master Mix (Yeasen Biotechnology, Shanghai, China), and 6.4 µL ddH_2_O, to reach a final volume of 20 µL. Primer sequences can be found in the Supplementary Materials (Table S6). The amplification was set as pre-denaturation at 95 °C for 2 min, 40 cycles including denaturation at 95 °C for 10 s, and extension at 60 °C for 30 s. The melting procedure followed the instrument’s default settings. The relative expression levels of genes were determined using the 2^−∆∆CT^ method.

### 4.9. Statistical Analysis

All experimental data were obtained from three replicates, and data are presented as mean ± standard error (SE). Significance differences in the data were evaluated using one-way analysis of variance (ANOVA). * *p* < 0.05, ** *p* < 0.01, *** *p* < 0.001, **** *p* < 0.0001. *p* < 0.05 (*) was considered statistically significant.

## 5. Conclusions

In this study, we have identified 10 *CcYTH* genes in camphor trees and conducted a comprehensive and systematic investigation of these m^6^A-binding proteins in the *C. camphora* genome, including phylogenetic analysis, chromosome localization, gene structure, and conserved motif and promoter analysis, as well as expression profiling. Our results demonstrate that the m^6^A-binding proteins in camphor trees exhibit a high degree of evolutionary conservation, especially within the woody plants. The identified genes exhibit tissue-specific expression patterns in leaves, stems, and roots. Additionally, the expression of *CcYTH* genes also undergoes changes in response to various abiotic stresses. Our findings establish a foundation for future functional analysis of these genes, providing a new perspective for future molecular breeding of *C. camphora* by applying epitransciptomic engineering.

## Figures and Tables

**Table 1:** Characteristics of CcYTH proteins family in *C. camphora*.

Gene Name	Gene ID	Locus	CDS (bp)	Protein Length (aa)	MW (kDa)	Aliphatic Index	GRAVY	pI	Subcellular Localization
*CcDC1A*	GWHGBGXC018157	Chr05	1827	608	66.7	61.64	−0.539	5.91	Nucleus
*CcDC1B*	GWHGBGXC008948	Chr02	1422	473	53.5	68.20	−0.617	8.62	Nucleus
*CcDF1A*	GWHGBGXC015419	Chr04	2118	705	76.9	60.26	−0.676	7.95	Nucleus
*CcDF1B*	GWHGBGXC020092	Chr05	2049	682	74.5	56.06	−0.618	5.63	Nucleus
*CcDF* *1* *C*	GWHGBGXC025982	Chr08	2160	719	80.7	59.64	−0.811	8.8	Cell membrane or Nucleus
*CcDF2C*	GWHGBGXC023812	Chr07	1764	587	64.9	60.29	−0.580	5.9	Nucleus
*CcDF* *3* *C*	GWHGBGXC017636	Chr04	1806	601	66.5	56.76	−0.676	5.42	Nucleus
*CcDF* *4* *C*	GWHGBGXC008047	Chr12	1791	596	65.8	61.16	−0.644	5.21	Nucleus
*CcDF* *5* *C*	GWHGBGXC005246	Chr11	2232	743	81.2	59.84	−0.655	6.64	Nucleus
*CcDF6C*	GWHGBGXC005110	Chr10	2040	679	74.3	73.03	−0.513	8.17	Chloroplast or Nucleus

## Data Availability

Data are contained within the article.

## Supplementary Materials

The following supporting information can be downloaded at: https://www.mdpi.com/article/10.3390/ijms25115960/s1.
